# Influenza Vaccination in Type 2 Diabetes Patients: Coverage Status and Its Determinants in Southwestern Saudi Arabia

**DOI:** 10.3390/ijerph15071381

**Published:** 2018-07-01

**Authors:** Ibraheem M. Alnaheelah, Nabil J. Awadalla, Khalid M. Al-Musa, Abdullah A. Alsabaani, Ahmed A. Mahfouz

**Affiliations:** 1 Joint Program for Postgraduate Studies in Community Medicine-Southern Region, Abha 61421, Saudi Arabia; ibr.asere@gmail.com (I.M.A.); drkalmosa@hotmail.com (K.M.A.); dr.alsabaani@hotmail.com (A.A.A.); mahfouz2005@gmail.com (A.A.M.); 2 Department of Family and Community Medicine, College of Medicine, King Khalid University, Abha 61421, Saudi Arabia; 3 Department of Community Medicine, Faculty of Medicine, Mansoura University, Mansoura 3551, Egypt; 4 Aseer General Directorate of Health Affairs, Ministry of Health, Abha 62523, Saudi Arabia; 5 Department of Epidemiology, High Institute of Public Health, Alexandria University, Alexandria 21511, Egypt

**Keywords:** diabetes, seasonal influenza vaccination, motivators and barriers, knowledge, determinants

## Abstract

Despite the significant role of seasonal influenza vaccination in preventing and minimizing the serious complications of influenza infection in type 2 diabetes mellitus (T2DM) patients, unsatisfactory compliance still exists for vaccination. Study objectives were to explore the vaccination status and determinants in T2DM patients in southwestern Saudi Arabia. A cross-sectional study on a representative sample of T2DM patients in Abha city, southwestern Saudi Arabia, was conducted. Data for sociodemographic characteristics, clinical criteria, vaccination status, vaccination motivators and barriers and seasonal influenza knowledge were collected. Out of 353 T2DM patients included in the study, seasonal influenza vaccination coverage was 61% in year 2017. A significant factors associated with non-vaccination were; poor influenza and its vaccine knowledge (OR = 4.31, 95% CI: 2.73–6.80), illiteracy (OR = 1.93, 95% CI: 1.11–3.37), and more than 10 years disease duration (OR = 2.07, 95% CI: 1.11–3.87). Presence of family history of DM and ischemic heart comorbidity minimized the possibility of non-vaccination (OR = 0.54 and 0.28 respectively). Healthcare givers’ advice was the most reported vaccination motivator (84.7%) while; fear of vaccine side effects was the most stated barrier (73%). In conclusion, influenza vaccination rate among T2DM in the present study is less than the recommended level. Continuous primary health care center-based educational programs should be implemented to aware and encourage influenza vaccination among T2DM patients.

## 1. Introduction

Globally, type-2 diabetes mellitus (T2DM) is a highly significant public health problem because of its health and economic impacts and its raising prevalence [1]. In Saudi Arabia, it affects about 46% of men and 44% of female in the “above 50 years” age group [2]. T2DM patients are at higher risk by six times to be hospitalized with influenza or pneumonia and three times more likely to die from influenza complications than others in general population [3].

Seasonal Influenza vaccination is the appropriate method to minimize the risk of death and hospitalization from influenza complications in T2DM patients [4].Therefore, annual influenza vaccination is recommended by the main diabetes associations to all patients with T2DM [5]. Even with the evident worth of seasonal influenza vaccine, influenza vaccination coverage is still low [6].

The motivators of influenza vaccination includes both personal and health status factors. These include; old age, good knowledge toward vaccine and presence of chronic disease. On the other hand fear from vaccine side effects and misimpression of vaccine inefficacy are the most reported barriers toward vaccination [4,5,7].

Saudi Arabia is one of the most important centers for international travel in the world. It received annually, millions of pilgrims and other visitors. Saudi ministry of health has implemented many policies to prevent the possible emerging or reemerging diseases associated with traveling. This includes vaccination for citizen and pilgrims with seasonal influenza vaccine [8].

Data about seasonal influenza vaccination coverage, personal and clinical determinants of influenza vaccination in Saudi Arabia in general and southwestern region in particular are scarce or even lacking. Identifying the extent of vaccination coverage and its determinants among T2DM patients in the region may be beneficial in resources allocations and primary healthcare policies development that might improve care of T2DM individuals.

The present study was conducted to explore the seasonal influenza vaccination coverage and determinants in T2DMpatients in Abha City, southwestern Saudi Arabia in the vaccination season 2017. 

## 2. Subjects and Methods

### 2.1. Study Area

Abha City is the capital of Aseer region and situated at an elevation 7500 feet above sea level. It included 11 Primary Health Care Centers (PHCCs) serving a population of 421,921. Aseer region is one of the largest regions in Saudi Arabia and located to the southwestern. 

### 2.2. Study Design and Target Population

A cross sectional study targeted male and female T2DM patients attending chronic disease clinics in the eleven PHCCs in Abha City. Chronic diseases clinics provide care for about 4324 diabetic patients regularly attending these centers. 

### 2.3. Sample Size Calculation and Sampling Technique

Using the World Health Organization manual for sample size determination in health study [9] with the total number of T2DM patients regularly attending the studied PHCCs being 4324 patients, a conservative anticipated proportion of 50% for vaccination coverage, and an absolute precision of 5% at a 95% confidence interval, the minimal sample size required for the study was calculated to be 353 subjects. Systematic random sampling with proportional distribution was used to collect the study subjects. The sample from each PHCC was calculated by multiplying the sample fraction with the number of diabetic patients regularly attending the chronic clinic of that center.

### 2.4. Data Collection

Data collected from the study T2DM patients who attended PHCCs during the 2017–2018 seasonal vaccination period (October through December 2017) by direct interview. Specially designed questionnaires was constructed using the Delphi technique after reviewing the relevant literature [10,11,12]. Experts in epidemiology, biostatisticians, and seasonal influenza vaccination programs validated the questionnaire. It includes the following data: (a) personal information such as, age, sex, education, and occupation; (b) clinical data such as the onset of T2DM, duration, compliance with management plans, complications and co-morbidities; (c) seasonal influenza vaccination status; (d) motivators and barrier of vaccination at this year; (e) five questions to assess influenza vaccine knowledge. Adequate knowledge was considered with the correct response to at least three questions.

### 2.5. Data Entry and Analysis

Collected data were entered, refined and analyzed by using SPSS, version 23 software package (IBM, North Castle, NY, USA). Chi square and odds ratio with a concomitant 95% confidence interval (95% CIs) were computed to assess the determinants of non-vaccination.

### 2.6. Ethical Approval

The study was carried out in accordance with the Declaration of Helsinki. Ethics and Research Committee of the College of Medicine of King Khalid University approved the protocol. Written approval was obtained from the directorate of Health sector in Aseer region and Abha City before starting the study. Oral consent was obtained from each participant after explaining the benefits of the study and guarantee for the confidentiality of collected data.

## 3. Results

The present study included 353 T2DM patients; Table 1 describes their influenza vaccination status in the vaccination season 2017–2018. More than half of the study group, (216 (61.2%)) were currently vaccinated or intended to be vaccinated. Most of the vaccinated group were male (131 (63%)) and vaccinated before (190 (88%)). About one-fifth (75 (21%)) of participants were found previously vaccinated and reject vaccination this year, while 17.6% of participants had never been vaccinated before. No statistically significant difference was found in vaccination status by gender.

Motivators and barriers for seasonal influenza vaccination in the study T2DM patients by gender are presented in Table 2. The most frequently reported motivators of seasonal influenza vaccination among included vaccinated or willing participants were advise from healthcare givers 183 (84.7%) and subject’s perception regarding the importance of vaccination (35.6%). Response to healthcare givers’ advise was significantly higher among female participants (*p* = 0.02). As for barriers, fear of vaccine side effects and the misconception that vaccine is not important or the vaccine could cause influenza like illness were the most frequently reported barriers (73%, 50.4%, and 30.7% respectively). Fear of needle shot and misconception that vaccine is not important were more frequently reported barriers in females (*p* < 0.05). Absence of healthcare givers’ recommendations and the difficult access to vaccine were the least reported barriers among unvaccinated group (8.8% and 0.7%).

Table 3 presents the univariate analysis for the personal factors potentially associated with seasonal influenza non-vaccination in the study T2DM participants. The risk of non-vaccination was significantly increased among illiterate (OR = 1.93, 95% CI: 1.11–3.37). On the other hand, the other personal factors were not significantly associated with non-vaccination among the study T2DM patients.

The univariate analysis for personal factors associated with seasonal influenza non-vaccination in the study of T2DM patients is presented in Table 4. The risk of non-vaccination was significantly higher among T2DM patients with above 10 years disease duration (OR = 2.07, 95% CI: 1.11–3.87), and having suffered from renal complications (OR = 4.15, 95% CI: 1.91–9.04). On the other hand, participants with a positive family history of diabetes (OR = 0.54, 95% CI: 0.53–0.85) and having suffered from ischemic heart disease co-morbidity (OR = 0.28, 95% CI: 0.08–0.98) were more prone to be vaccinated. 

More than half of the study participants (55%) were adequately knowledgeable about influenza. Inadequate knowledge was significantly higher in age group above 50 years old, duration of T2DM of more than 10 years and absence of positive family history of diabetes. The risk of non-vaccination was significantly higher (OR = 4.31, 95% CI: 2.73–6.80) among those who lack adequate knowledge (data not tabulated).

## 4. Discussion

The results of the current study provide insight into vaccination status with seasonal influenza vaccine amongT2DM patients for the vaccination season 2017–2018. It confirms that more than half (61%) of T2DM patients are vaccinated. This figure is very close to the coverage rate of vaccination among Korean diabetic patients which amounted to 57.7% [13] and higher than that in Spain (40%) [14] and France (33.7%) [15]. The current governmental and public interest about respiratory infectious diseases and corona virus in Saudi Arabia [8,16] besides the free and easy access to influenza vaccine are possible reasons for the increased seasonal influenza coverage compared to other world areas that reported low coverage rate for seasonal influenza vaccine.

Our results revealed that the healthcare givers’ advice is the most important motivating factor for vaccination among T2DM participants. The healthcare givers play an important role in clarifying the necessity for vaccination to the health of their patients and encouraging them for vaccination [15,17,18]. On the other hand, according to our study, media advice for vaccination cams as the least motivating factor. This may be explained by either lack of interest in following media among elders or due to absence of clear educational message for influenza vaccination delivered by the media [19].

In the current study, fear from side effect of vaccination was commonest barrier among participants at this year while, difficult access to vaccine was the least barrier. In Saudi Arabia, influenza vaccination is accessible and given free of charge to all T2DM patients attending the PHCCs. Therefore, our results confirmed the existence of some influenza vaccine related misconceptions as “vaccine could cause side effects”. Similarly, previous studies have confirmed that lack of correct knowledge about influenza vaccine is an important obstacle to vaccine recipients [15,18,20,21].

Our study suggests that the risk of seasonal influenza non-vaccination is significantly higher among illiterate T2DM patients. This result is in accordance with studies conducted in Saudi Arabia [20], Spain [22], and the United States [23]. The educational status significantly affects the health and wellbeing of the individuals. Educated participants are more able to receive, understand, and be motivated by seasonal influenza vaccination messages compared with illiterates [24]. In the same context, the current study observed that T2DM patients with inadequate influenza knowledge are at higher risk of non-vaccination compared with adequately knowledgeable patients. This result is in agreement with a recent South African study that found that T2DM patients with good influenza knowledge were 3.8 times more prone to be vaccinated compared with those with poor knowledge [25]. Therefore, the insufficient knowledge and misconceptions result in low seasonal influenza vaccine uptake [4].

Among the high-risk groups that should be vaccinated against seasonal influenza is T2DM patients with real complication [23].

The results of the presents study revealed that, the risk of non-vaccination was significantly higher among T2DM patients with above 10 years disease duration. This finding is in inconsistent with the studies conducted among diabetic patients in France [15], Spain [12], and south Africa [25], which reported a positive association between duration of diabetes and influenza vaccine uptake. The possible explanation of this controversy may be related to the indirect effect of inadequate influenza knowledge observed in the current study among elders and patients with a duration of diabetes of more than 10 years.

Presence of renal complication was observed as a significant factor positively associated with non-vaccination. This find is against the influenza vaccine recommendations for diabetic patients, especially those who developed renal or cardiac complications. There is no evidence that vaccination with seasonal influenza vaccine could worsen the impaired renal functions caused by diabetic nephropathy [26,27]. The limited vaccine uptake among those with renal complications in the current study could be attributed to the misconception of the patients or their healthcare givers about the adverse effects of the vaccine on renal functions [4].

The present study found a higher influenza vaccination rate among T2DM patients with a positive family history of diabetes. This could be attributed to the higher level of influenza knowledge observed in the current study among patients with positive family history of diabetes. 

The finding of the current study confirms previous studies conducted in Spain [12] and Canada [28] which reported a positive association between having ischemic heart comorbidity and influenza vaccination among T2DM patients. A meta-analysis study has concluded that influenza vaccination reduced the probability of major cardiovascular complications in patients with coronary artery disease in comparison with no vaccination [29].

The present study gives new and important information regarding the influenza vaccine coverage and its associated determinants, motivators and barriers in T2DM patients attending PHCCs in southwestern Saudi Arabia. The experiences gained might be generalized in comparable populations to increase influenza vaccination coverage to minimize the risk of influenza among T2DM patients. Yet, the study has some limitations related to its design. These include; the study population may not be representative to all T2DM patients as we included only those who attending PHCCs. Second, the study may be liable for information recall bias. Third, the study variables not included healthcare givers’ knowledge and attitude toward influenza vaccination in T2DM patients which may affect participants’ uptake and could act as possible confounders.

## 5. Conclusions

In conclusion, influenza vaccination among T2DM patients attending PHCCs in Southwestern Saudi Arabia is suboptimal. Insufficient knowledge and misconceptions about influenza and its vaccine are the main barriers to vaccination while, healthcare givers’ advice is the main motivator. Patients with a positive family history of diabetes, education, and suffering from ischemic heart disease comorbidity are more prone to vaccine uptake. Continuous PHCCs-based health education programs with different approaching methods should be conducted to educate T2DM patients and motivate them toward influenza vaccination.

## Figures and Tables

**Table 1:** Influenza vaccination status in vaccination season 2017–2018 among study type 2 diabetes mellitus (T2DM) patients by gender (*N* = 353).

Seasonal Influenza Vaccination Status	Total (*N* = 353) No %	Male (*N* = 208) No %	Female (*N* = 145) No %	*p* Value
Previously vaccinated but not this season	75 (21.0)	40 (19.2)	35 (24.1)	0.27
Currently vaccinated	216 (61.2)	131 (63.0)	85 (58.6)	0.41
Never vaccinated	62 (17.6)	37 (17.8)	25 (17.2)	0.89

**Table 2:** Motivators and barriers for seasonal influenza vaccination in the study T2DM patients by gender.

	Total No %	Male No %	Female No %	*p* Value
**Motivators for Currently Vaccinated or Intended (*N* = 216) ^#^**				
Vaccine is important	77 (35.6)	50 (38.2)	27 (31.8)	0.34
Healthcare givers advise	183 (84.7)	105 (77.8)	78 (91.8)	0.02 *
Peers or relatives advise	28 (13.0)	13 (9.9)	15 (17.6)	0.10
Media advise	27 (12.5)	16 (12.2)	11 (12.9)	0.91
**Barriers for Currently Non Vaccinated (*N* = 137) ^#^**				
Vaccine is not recommended	12 (8.8)	9 (11.7)	3 (5.0)	0.18
Vaccine is not important	69 (50.4)	45 (58.4)	24 (40.0)	0.04 *
Fear of side effects	100 (73.0)	56 (72.7)	44 (73.3)	0.84
Fear of needle shot	21 (15.3)	7 (9.1)	14 (23.3)	0.02 *
Vaccine cause influenza illness	42 (30.7)	22 (28.6)	20 (33.3)	0.52
Difficult to access	1 (0.7)	0 (0.0)	1 (1.7)	0.25
Influenza isn’t serious disease	31 (22.6)	19 (24.7)	12 (20.0)	0.54

^#^ Data is not mutually exclusive, * Significant (*p*-value ≤ 0.05).

**Table 3:** Univariate analysis for personal factors associated with seasonal influenza non-vaccination in the study T2DM patients (*N* = 353).

Personal Factors	Vaccinated (*N* = 216) No %	Non Vaccinated (*N* = 137) No %	OR (95% CI)
**Age (years):**			
<50	68 (68)	32 (32)	0.66 (0.41–1.85)
>50	148 (58.5)	148 (58.5)	Ref.
**Sex:**			
Male	131 (63)	77 (37)	0.83 (0.54–1.29)
Female	85 (58.6)	60 (41.4)	Ref.
**Nationality:**			
Saudi	210 (61.4)	132 (38.6)	0.75 (0.23–2.52)
on-Saudi	6 (54.5)	5 (45.5)	Ref.
**Marital status:**			
Married	172 (63.9)	97 (36.1)	0.62 (0.38–1.02)
Single	44 (44.1)	40 (55.9)	Ref.
**Level of education**			
Illiterate	64 (52)	59 (48)	**1.93 (1.11–3.37)**
Lower than secondary school	87 (64.9)	47 (35.1)	1.13 (0.65–1.97)
Secondary school or above	65 (67.7)	31 (32.3)	Ref.
**Occupation**			
Employee	53 (70.7)	22 (29.3)	0.78 (0.41–1.45)
Business work	6 (50.0)	6 (50.0)	1.89 (0.57–6.28)
Housewife/Unemployed	89 (54.9)	73 (45.1)	1.55 (0.93–2.58)
Retired	68 (65.4)	36 (34.6)	Ref.
**Monthly income**			
Sufficient and exceed	9 (64.3)	5 (35.7)	0.98 (0.30–3.24)
Sufficient	161 (60.3)	106 (39.7)	1.16 (0.68–2.00)
Insufficient	46 (63.9)	26 (36.1)	Ref.
**Smoking status**			
Current smoking	16 (59.3)	11 (40.7)	1.14 (0.51–2.55)
Ex-smoker	21 (53.8)	18 (46.2)	1.42 (0.72–2.79)
Never	179 (62.4)	108 (37.6)	Ref.

OR = Odds Ratio, 95% CI = 95% Confidence Interval, Bold 95% CIs is statistically significant, Ref. = Reference group.

**Table 4:** Univariate analysis for clinical factors associated with seasonal influenza non-vaccination in the study T2DM patients (*N* = 353).

Clinical Factors	Vaccinated (*N* = 216) No %	Non Vaccinated (*N* = 137) No %	OR (95% CI)
**Duration of DM (years)**			
<5	49 (73.1)	18 (26.9)	Ref.
5–10	78 (60.9)	50 (39.1)	1.71 (0.89–3.27)
>10	89 (56.3)	69 (43.7)	**2.07 (1.11–3.87)**
**Family history of DM**			
Yes	153 (66.2)	78 (33.8)	**0.54 (0.53–0.85)**
No	63 (51.6)	59 (48.4)	Ref.
**Type of DM treatment**			
Oral	129 (59.4)	88 (40.6)	1.20 (0.73–1.99)
Insulin	27 (64.3)	15 (35.7)	0.98 (0.46, 2.09)
Both	60 (63.8)	34 (36.2)	Ref.
**Compliance with treatment**			
Good	145 (59.2)	100 (40.8)	0.64 (0.38–1.08)
Moderate	61 (69.3)	27 (30.7)	0.44 (0.17–1.19)
Poor	10 (50)	10 (50)	Ref.
**Compliance with follow up**			
Good	120 (60.6)	78 (39.4)	0.69 (0.33–1.42)
Moderate	78 (65)	42 (35)	0.57 (0.27–1.22)
Poor	18 (51.4)	17 (48.6)	Ref.
**Compliance with physical activity**			
Good	35 (63.6)	20 (36.4)	0.88 (0.47–1.62)
Moderate	60 (61.2)	38 (38.8)	0.97 (0.59–1.59)
Poor	121 (60.5)	79 (39.5)	Ref.
**Compliance with diet regimen**			
Good	45 (62.5)	27 (37.5)	1.20 (0.79–1.82)
Moderate	103 (56.6)	79 (43.4)	1.68 (1.00–2.82)
Poor	68 (68.7)	31 (31.3)	Ref.
**Diabetic complication**			
Renal	10 (30.3)	23 (69.7)	**4.15 (1.91–9.04)**
Visual	65 (61.3)	41 (38.7)	0.99 (0.62–1.58)
Peripheral neurovascular	5 (45.5)	6 (54.5)	1.27 (0.33–4.81)
**Comorbidities**			
Bronchial asthma	7 (46.7)	8 (53.3)	1.85 (0.65–5.22)
Ischemic Heart Disease	16 (84.2)	3 (15.8)	**0.28 (0.08–0.98)**
Hypertension	40 (50.6)	39 (49.4)	1.75 (1.00–2.90)
**Last year hospitalization**			
Yes	19 (67.9)	9 (32.1)	0.73 (0.32–1.66)
No	197 (60.6)	128 (39.4)	Ref.
Diabetic control (HbA1c) *			
≤7%	22 (71)	9 (29)	0.94 (0.39–2.25)
>7%	78 (69.6)	34 (30.4)	Ref.

OR = Odds Ratio, 95% CI = 95% Confidence Interval, Bold 95% CIs is statistically significant, Ref. = Reference group, * out of 143 cases.
